# Sequencing strategy for the whole mitochondrial genome resulting in high quality sequences

**DOI:** 10.1186/1471-2164-10-139

**Published:** 2009-03-30

**Authors:** Liane Fendt, Bettina Zimmermann, Martin Daniaux, Walther Parson

**Affiliations:** 1 Institute of Legal Medicine, Innsbruck Medical University, Müllerstrasse 44, Austria; 2 Clinical Department of Radiology, Innsbruck Medical University, Austria

## Abstract

**Background:**

It has been demonstrated that a reliable and fail-safe sequencing strategy is mandatory for high-quality analysis of mitochondrial (mt) DNA, as the sequencing and base-calling process is prone to error. Here, we present a high quality, reliable and easy handling manual procedure for the sequencing of full mt genomes that is also appropriate for laboratories where fully automated processes are not available.

**Results:**

We amplified whole mitochondrial genomes as two overlapping PCR-fragments comprising each about 8500 bases in length. We developed a set of 96 primers that can be applied to a (manual) 96 well-based technology, which resulted in at least double strand sequence coverage of the entire coding region (codR).

**Conclusion:**

This elaborated sequencing strategy is straightforward and allows for an unambiguous sequence analysis and interpretation including sometimes challenging phenomena such as point and length heteroplasmy that are relevant for the investigation of forensic and clinical samples.

## Background

Investigations of the human mt genome are in the focus of biological and medical scientific disciplines. Compared to nuclear DNA (nDNA), mitochondrial DNA (mtDNA) is more vulnerable to oxidative damage and undergoes a higher rate of mutation [1]. Because of these features the analysis of the mt genome has become a proven tool in population genetics. A multi-copy genome without recombination which accumulates mutations allows for the establishment of phylogenetic trees [2]. It was the information from the highly variable mitochondrial control region (CR) that lifted the secret of human evolution starting in Africa about 150000 years ago and gave an insight in human migration all over the world within the past 60000 years [3,4]. Sequences of full mt genomes are necessary to decipher yet not defined haplotypes and assign them to their phylogeographic environment.

Mitochondrial DNA mutations in the coding region (codR) have been associated with several pathologies [5] including cancer [6-9]. During oxidative phosphorylation (OXPHOS) mitochondria produce reactive oxidative species (ROS) that potentially induce DNA mutations. Such an initial mutation is heteroplasmic with the mutated variant constituting a minority [10]. In the course of several replications the heteroplasmic mutation may become dominant leading to cancer [8]. This theory is based on the results of several investigations on cancer tissues [11-18]. Unfortunately, numerous articles addressing that issue are erroneous as reviewed in [19,20]. On the one hand it is the lack of phylogenetic knowledge and the ambiguous mtDNA alignment that led to false conclusions of mtDNA mutations to be tumor-specific rather than evolutionary caused. On the other hand, laboratory-, sequencing-, and analysis errors led to wrong base-calls [21]. Hence, flawed data hamper a precise interpretation of the conjunction between mtDNA mutations and the complex process of tumor development.

For forensic as well as for phylogenetic purposes we have already successfully established evaluated sequencing strategies that proved to be useful in a number of investigations where precise base-calling was necessary for the CR [22-26], however such stringency is lacking for the whole mt genome. The published protocols vary concerning the number and sizes of PCR products, the chemistry employed, and the number of sequencing primers [27-31]. One review reports the use of 58 sets of unique sequencing primers to completely cover the mt genome, while another protocol provides 77 sequencing primers for the codR and 7 additional primers for the CR [27]. There, sequencing is performed on 12 amplicons that cover the whole mt genome in an overlapping manner [29]. In a recent protocol [28] the amplification of the entire mt genome was conducted with only two overlapping amplicons, followed by 48 upstream and downstream sequencing reactions. Whereas amplicon sizes must be kept short for forensic samples for reasons of limited DNA quality and quantity, a reduction of the necessary amplicons is desirable for other applications, where usually fresh DNA is obtained. This simplifies the laboratory work and minimizes potential amplicon mix-up [19]. Independent of the amplification strategy high sequence quality is required to achieve reliable base-calling.

We addressed this issue by presenting a set of 96 carefully selected sequencing primers that are embedded in a reliable and fail-safe sequencing strategy. The following criteria were applied to guide the development. (1) Each nucleotide reported in the consensus sequence should derive from at least two independent sequencing reactions using different primers (double strand coverage) to avoid the reporting of phantom mutations and other ambiguous base-callings. (2) We envision a minimum number of PCR products to reduce the chance for amplicon mix-up during the (manual) set-up of sequencing reactions and (3) we selected primers that produce sequences with an optimal signal-to-noise ratio to enable unequivocal assignment of point and length heteroplasmy.

## Results

### Methodical procedure

**Figure 1:**
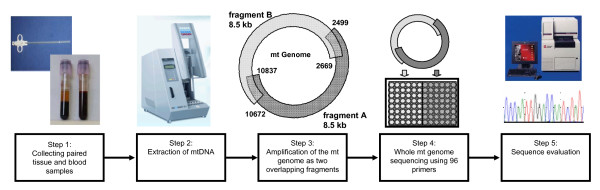
**Overview of the laboratory and analysis procedure**. Tissue and blood samples were extracted using the EZ1 biorobot (Qiagen, step 2). Mt genomes were amplified as two overlapping fragments A and B (step 3), which were added to the respective pre-pipetted sequencing primers (step 4). Cycle sequencing products were analyzed by capillary electrophoresis (3100 Applied Biosystems, step 4).

The complete mtDNA was amplified using 2 overlapping fragments each about 8.5 kb in length with primers published in [28]. Fragment A ranges within nucleotide positions 2499 and 10837, fragment B between 10672 and 2669 comprising the CR (Table 1, Figure 1, step3).

**Table 1:** List of primers for amplification and full double-stranded sequence coverage of the entire mtDNA codR

	**Name**	**Sequence 5'**	**Corresponding fragment**
PCR	FampA	AAATCTTACCCCGCCTGTTT	A
PCR	RampA	AATTAGGCTGTGGGTGGTTG	A
PCR	FampB	GCCATACTAGTCTTTGCCGC	B
PCR	RampB	GGCAGGTCAATTTCACTGGT	B

1	F1	CCGCTTCTGGCCACAGCACT	B
2	F2	GGTTGGTCAATTTCGTGCCAG	B
3	R1	ACTTGGGTTAATCGTGTGACC	B
4	F3	CATCAAGCACGCAGCAATG	B
5	F4	CTCACCACCTCTTGCTCAGC	B
6	F5	CTTGACCGCTCTGAGCTAAAC	B
7	F6	AAGCTAAGACCCCCGAAACC	B
8	F7	AAACCTACCGAGCCTGGTG	B
9	F8	GAGGAACAGCTCTTTGGACAC	B
10	F9	TCGTCCCAACAATTATATTACTACCA	B
11	R2	CTGTTTGTCGTAGGCAGATGG	B
12	F10	AACGCCACTTATCCAGTGAACC	B
13	F11	GACTCCCTAAAGCCCATGTCG	B
14	F12	CATCTGCCTACGACAAACA	B
15	F13	ACAGCCATTCTCATCCAAACCC	B
16	F14	AACCACGTTCTCCTGATCAAA	B
17	R3	GATATCGCCGATACGGTTG	B
18	R4	AGCGGATGAGTAAGAAGATTCC	B
19	R5	TTGAAGAAGGCGTGGGTACAG	B
20	F15	TTCATCCCTGTAGCATTGTTCG	B
21	F16	TTGCTCATCAGTTGATGATACG	B
22	F17	CACTCTGTTCGCAGCAGTATG	B
23	F18	CATCATCGAAACCGCAAAC	B
24	F19	TTTCTCCAACATACTCGGATTC	B
25	F20	ACAAACAATGGTCAACCAGTAAC	B
26	F21	TCCAAAGACAACCATCATTCC	B
27	R6	TTATCGGAATGGGAGGTGATTC	B
28	F22	TACTCACCAGACGCCTCAACCG	B
29	F23	AGTCCCACCCTCACACGATTC	B
30	F24	CGCCTACACAATTCTCCGATC	B
31	R7	CGGTTGTTGATGGGTGAGTC	B
32	F25	AAATGGGCCTGTCCTTGTAG	B
33	R8	TCATAAGGGCTATCGTAGTTTTC	B
34	F26	GTGGCAAGAAATGGGCTAC	B
35	F27	AACATATAACTGAACTCCTCACACC	B
36	F28	GCCGCAGTACTCTTAAAACTAGG	B
37	F29	AGGACTCAACATACTAGTCACAGC	B
38	F30	GCCATACTAGTCTTTGCCGC	B
39	R9	GCTGTGTTGGCATCTGCTC	B
40	F31	AAAGACCACATCATCGAAACC	B
41	F32	CTAACAGGTCAACCTCGCTTCC	B
42	F33	CCTTCATAAATTATTCAGCTTCCT	B
43	F34	CAATGATATGAAAAACCATCGTT	B
44	R10	GGATGGCGGATAGTAAGTTTGT	B
45	F35	CAGGGTTGGTCAATTTCGT	B
46	F36	AATGGTTTGGCTAAGGTTGT	B
47	R11	ACGAACAATGCTACAGGGATG	B
48	F37	GGCATTATCCTCCTGCTTGCAACTAT	B
49	R12	ATGTCCTGATCCAACATCGAG	A
50	R13	AGAAGAGCGATGGTGAGAGC	A
51	F38	CGACCTCGATGTTGGATCAGGACA	A
52	F39	AGATGGCAGAGCCCGGTAATC	A
53	F40	ACTACAACCCTTCGCTGACG	A
54	F41	CCCTAGCATTACTTATATGATATGTCTCCATACCCATTACAATCTCC	A
55	F42	TCAGGCTTCAACATCGAATACG	A
56	F43	CCCATCCTAAAGTAAGGTCAGC	A
57	F44	CCCTTTCACTTCTGAGTCCCAG	A
58	F45	CACCATCACCCTCCTTAACC	A
59	R14	GCTGAGTGAAGCATTGGACTG	A
60	F46	TAAGCACCCTAATCAACTGGC	A
61	R15	ATAGTGATGCCAGCAGCTAGG	A
62	F47	CGCATCTGCTATAGTGGAGG	A
63	R16	TTTCATGTGGTGTATGCATCG	A
64	F48	GCCATAACCCAATACCAAACG	A
65	F49	GAGGCTTCATTCACTGATTTCC	A
66	R17	GGGCAGGATAGTTCAGACGG	A
67	F50	TTCCCACAACACTTTCTCGGCC	A
68	R18	AAGTTAGCTTTACAGTGGGCTCTAG	A
69	F51	CGGTCAATGCTCTGAAATCTGTG	A
70	F52	CTGTTCGCTTCATTCATTGCC	A
71	R19	GTGGCGCTTCCAATTAGGTG	A
72	R20	GTGCTTTCTCGTGTTACATCG	A
73	R21	GAAAGTTGAGCCAATAATGACG	A
74	F53	TTTCACTTCCACTCCATAACGC	A
75	F54	CCTGATACTGGCATTTTGTAGATGTGG	A
76	F55	ACTACCACAACTCAACGGCTAC	A
77	F56	CTAACCGTGCAAAGGTAGCA	A
78	F57	GCAATTCCCGGACGTCTAAACCAAA	A
79	F58	GCCATAATATGATTTATCTCCACA	A
80	F59	AAACCCTCGTTCCACAGAA	A
81	F60	GATGAATAATAGCAGTTCTACCGT	A
82	F61	CAACGTAAAAATAAAATGACAGTT	A
83	F62	ATATGAAAATCACCTCGGAGC	A
84	R22	AGTTACAATATGGGAGATTATTCC	A
85	F63	CGCAAGTAGGTCTACAAGACG	A
86	F64	CTAATCTTCAACTCCTACATACTTCC	A
87	R23	ATCTGTTTTTAAGCCTAATGTGG	A
88	F65	AAGATTAAGAGAACCAACACCTCT	A
89	F66	AACAACCGACTAATCACCACCCAACAATG	A
90	F67	TCATCTTCACAATTCTAATTCTACTG	A
91	F68	TCGAGTCTCCCTTCACCATT	A
92	F69	CGGCTTCGACCCTATATCC	A
93	R24	GGTAAAAGGAGGGCAATTTCT	A
94	F70	CTACTCTCATAACCCTCAACACC	A
95	F71	ATTAAACCAGACCCAGCTACG	A
96	F72	AGCATATTTCACCTCCGCTAC	A

**Figure 2:**
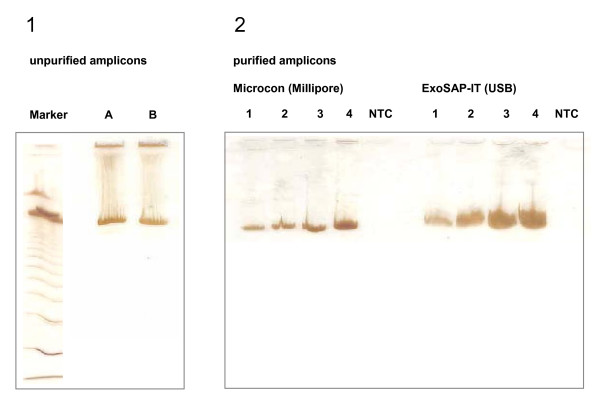
**PCR products of fragments A and B assessed in a polyacrylamide gel**. (1) Amplification products of unpurified fragments A and B (each 5000 genomic equivalents). (2) Fragment A purified with filter plates (Microcon (Millipore), left side) and digestion (ExoSAP-IT USB, right side). PCR template amounts were 1000 (lane 1), 2500 (lane 2), 5000 (lane 3) and 10000 (lane 4) mtDNA genomic equivalents.

**Figure 3:**
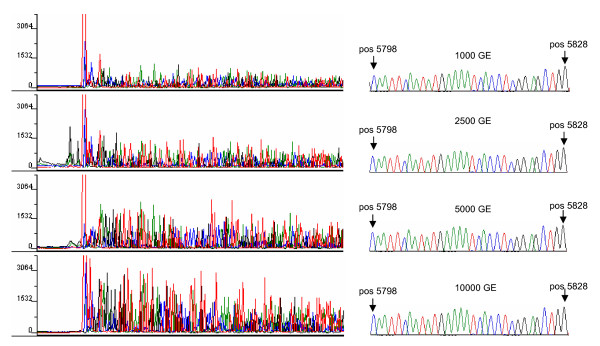
**Sequence analysis of different mtDNA dilutions**. left: raw data and (right) electropherograms of the sequencing reactions: amplification was carried out using 1000, 2500, 5000 and 10000 genome equivalents (GEs) of mtDNA using 2 μL of purified (Microcon) PCR products for cycle sequencing. Sequence electropherograms of fragment A from position 5798 to position 5828 are shown as example. Sequences of amplicons purified with ExoSAP-IT are not displayed as they equal with respect to the quality of the purified amplicons using filtration.

The sensitivity of the amplification reaction was elicited with 1000, 2500, 5000, and 10000 molecules of quantified mtDNA [32]. PCR yield and specificity were visualized by polyacrylamide gel electrophoresis (Figure 2). Even though the density of the banding pattern varied significantly (depending on the amplified DNA amount), the sequence analysis of all fragments resulted in comparably clear data (Figure 3, right). Also the amount of 1000 mtDNA genomic equivalents (GEs) that roughly corresponds to the mtDNA content of a single lymphocyte (fresh sample) proved sufficient for reliable results (given that the mtDNA is intact). PCR products showed light smear on the polyacrylamide gel (Figure 2), especially with increasing template amount. However, we did not observe negative effects on the quality of the sequencing results (Figure 3). No evidence of contamination was observed, neither in the non-template controls nor during evaluation of the individual mtDNA haplotypes. Both extraction blanks and PCR negative controls were free of signal. The mtDNA of two tissues (breast and blood) was extracted, amplified and sequenced from each person at different times and corresponding haplotypes gave a match after comparison (the haplotypes did not match laboratory staff).

Amplicons were purified according to two different protocols. We compared an enzymatic digestion method with a filtration method (Figure 2). We did not observe significant differences with respect to the DNA amount after purification except for one sample. There, the banding signal of the ExoSAP-IT purified product was less dense as compared to the filtrated one. However, despite of the different banding intensities (Figure 2) we did not observe relevant changes in sequencing quality between the electropherograms (Figure 3). The filtration method has the appealing advantage that it is less expensive for PCR product purification of few samples present in large volumes (up to 0.5 ml). One Microcon column is needed for each sample irrespective of the DNA volume to be added, whereas the required amount of ExoSAP-IT is required to be proportional to the volume of DNA (2 μL per 5 μL PCR-product).

**Figure 4:**
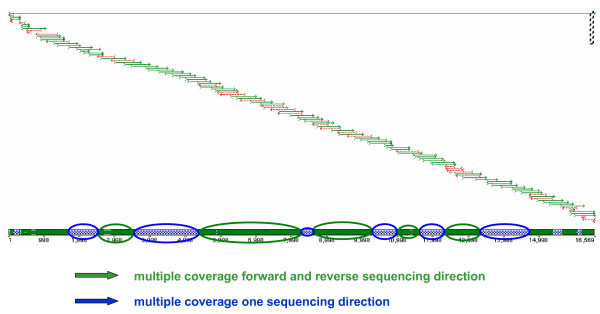
**Alignment of 96 sequence strands covering the mtDNA codR**. Multiple sequence coverage is indicated in blue color for regions with sequences of equally oriented primers whereas green areas indicate multiple coverage derived from forward and reverse sequencing reactions. Ten control region sequences [36] were added to the contig to complete the full mt genome sequence.

**Figure 5:**
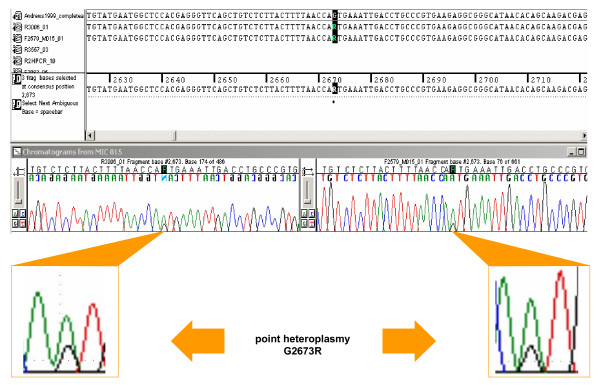
**Example of a sequence electropherogram**. The sequences of forward primer F56 reading 3' of nucleotide position 2579 and of reverse primer R12 reading 5' of position 3006 embrace point heteroplasmy at nucleotide position 2673 in the ND2 gene.

We established a set of 96 sequencing primers that lead to full double-sequence coverage of the complete mtDNA codR (Figure 4). Primer sequences were partly taken from [29,33] and  as well as designed inhouse [34]. In combination with sequences obtained from the CR [35] this resulted in high-quality sequence information for the whole mtDNA genome. Such a strategy makes sense, as full mt genome sequencing is usually carried out on selected samples that have earlier been analyzed within the CR. Sequencing was performed on the basis of the Big Dye Terminator Ready Reactions Kit protocol (Applied Biosystems, Foster City, CA). The quality of the primers was generally stable despite of multiple freezing/thawing cycles of the stock primer plate. For 6 primers (R4, R7, R18, R20, R21, F14) out of 96 the signal-to-noise ratio decreased after the fifth freezing/thawing cycle. An example of the general quality of the sequences is shown in Figure 5. The sequence is displayed by two primers in both directions confirming point heteroplasmy of the variants A and G at position 2673 within the NADH dehydrogenase (ND2) gene.

Sequence electropherograms did not differ substantially in terms of the signal-to-noise ratio as well as with respect to the relative peak heights when comparing the tested mtDNA template amounts. Although a relative increase of the fluorescence signal in the raw data was detected (Figure 3, left), the quality of the sequence electropherograms were comparable (Figure 3, right). In this study we tested template mtDNA amounts between 1000 and 10000 GEs that turned out to cover an appropriate template amount range for long PCR fragments.

### Data analysis and quality assurance

Upon analysis of the raw data the sequences were aligned and the base-calls reviewed twice by two independent scientists, such as has been found invaluable for CR analysis [35]. Consensus sequences were aligned and compared to the revised Cambridge Reference Sequence (rCRS) [36,37] following nomenclature guidelines for mtDNA typing [38-40]. In an independent analysis the two consensus sequences underwent comparison by means of a dedicated in-house software [41,42]. This concept enabled full electronic data handling minimizing the risk to introduce clerical errors.

### Assignment of the samples to their specific haplogroups

**Figure 6:**
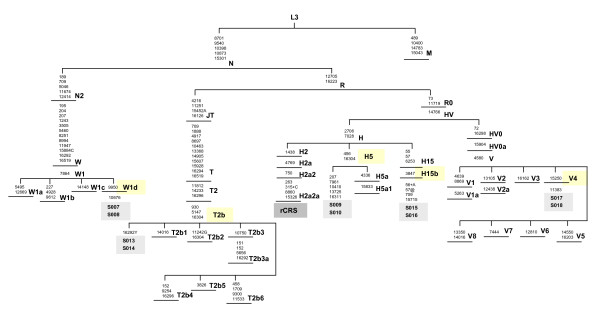
**Excerpt of the West Eurasian phylogeny**. The samples we tested in this study were assigned to the following West Eurasian haplogroups and are highlighted: samples S007/008: W1d, samples S009/010: H5, samples S013/S014: T2b, samples S015/S016: H15b samples S017/S018: V4. Position numbers refer to the rCRS [37] and designate transitions unless a suffix indicates a transversion (A, C or T) and an insertion (+). Back mutations are marked with @.

We present the complete mtDNA sequences of 10 clinical samples from 5 patients with respect to the phylogeny [43] (Figure 6) [sequences were deposited in GenBank  with accession numbers FJ384431–FJ384440]. These patients were classified as typical West-Eurasian lineages as members of haplogroups W1d, T2b, V4, H5, and H15b [33,44,45]. As shown in Figure 6 (highlighted in light grey), the mtDNA sequences generated from the primary cancerous tissue and the peripheral blood cells were identical in all 5 patients.

## Discussion

We present a reliable laboratory strategy for the amplification and sequence analysis of the full mt genome that meets a high quality demand. The method is conceived for samples that include an adequate amount and quality of DNA, such as found in fresh clinical and forensic samples and can be conducted by any laboratory in the molecular field without the requirement for automated liquid handling devices. The complexity of the laboratory concept is low, so that handling errors and risk of sample mix-up and contamination are minimized.

### Effects of primer-storage on sequencing quality

It has been shown in this study that the sequence performance of the majority of primers was not affected by different storage conditions. Only 6 primers (R4, R7, R18, R20, R21, F14) suffered from a treatment of more than 5 freezing/thawing cycles. Temperature changes caused a diminished function visualized by a disadvantageous signal-to-noise ratio depicted in the electropherogram. The ability to correctly identify point heteroplasmy strongly depends on the quality of the overall signal height and the signal-to-noise ratio. Consequently, an apparent improvement of the storing conditions would be the generation of stock plates including less primer volume sufficient for about 5 sequencing reactions. Alternatively, sequencing primers could be kept in a low tris buffer at 4°C.

### Applications in various fields

Recent research has been addressing the role of mitochondria and the mtDNA in aging and cancer as mitochondria participate in fundamental processes of the cellular metabolism [46]. The mitochondrial theory of aging implies that tissue function sustains until the number of cells declines below a threshold. The time range required to reach this threshold is related to the rate at which mitochondrial and mtDNA damage accumulates. If the mitochondrial ROS production rate increases, the rate of cell loss will also increase, resulting in early tissue failure and age-related disease.

Not only aging processes but also carcinogenesis have been linked to mutation based mitochondrial dysfunction since a general feature of tumor biology is the impaired energy metabolism [47]. For those investigations addressing aging and tumor biology, mitochondrial genes encoded in the nucleus and in the mitochondrial DNA are being analyzed. Genes encoded in the mitochondrial DNA can be systematically targeted with this evaluated sequencing strategy.

Furthermore, it can be helpful for population genetic and forensic applications where further information to the CR is required. In the forensic context this is particularly relevant for the few CR haplotypes that are shared more commonly within a population. In the West Eurasian population this is the case for the CR haplotypes 16519C 263G 315.1C and 263G 315.1C, which occur at frequencies of 0.013 and 0.002, respectively [48]. When such haplotypes are observed in a forensic case between suspect and crime scene samples one needs to consider the possibility that the sequences are identical by state and not by descent. Especially, as it is known that these haplotypes occur in more haplogroup backgrounds, such as H1, H2a, H3, H7, H10, H13a1, HV, R0 and the great bulk of yet unidentified sub-H lineages [26,49]. To increase the information content of such haplotypes we have earlier introduced a screening method based on single-base-extension reactions that target informative SNPs (single nucleotide polymorphism) in the codR [50]. Such screening methods are a valuable supplement to standard mtDNA sequencing as they generally increase the discrimination power and indicate the hg-affiliation of an mtDNA haplotype [49]. The logical continuation of this strategy would be the analysis of entire mt genomes in forensic casework for achieving maximum discrimination power. The current sequencing technology allows that only for relatively high sample qualities, but it is a first step towards mt genome sequencing in forensics.

In the population genetic field an accurate deciphering of the human mitochondrial phylogeny can only be conducted on the basis of full mt genomes. As a general approach full genome mtDNA sequencing is carried out on a defined selection of CR sequences as evidenced by recent examples [51,52].

### Meaning of obtaining high quality sequences

The focus of this strategy lies on applications where secure base-calling and high quality sequence data are mandatory for interpretation. It is well known that mitochondrial mutations associated to disease such as mitochondrial encephalomyopathies occur in heteroplasmic status and that the severity and the progression of the syndrome depend on a threshold above which the mutant triggers the pathological pathway [53,54]. The contribution of mtDNA mutations to carcinogenesis underlies the same assumption. It appears evident that particular mitochondrial defects with functional consequences exhibit an advantage in tumor development only if a certain threshold of mutated mtDNA populations is achieved. All the more it is important to pinpoint the relative quantity of these heteroplasmic mutations to estimate functional consequences of the genes involved. Moreover, good sequencing quality is the prerequisite to distinguish early stage point heteroplasmy from signal background which might be an issue in early stage cancer detection [55].

The occurrence and the frequency of mtDNA control region (CR) point heteroplasmy is an important issue in forensic case work [56] as the detection of point heteroplasmy, especially at low level, increases the power of discrimination between sequences [57].

## Conclusion

The codR sequencing method described herein is an optimized protocol that can also be applied in laboratories that do not have automated processes available. The overall aim, namely the achievement of a secure base-calling method was accomplished by the assortment of primers that allow for full double-stranded sequence coverage of the whole mtDNA genome (in combination with previously described CR sequencing strategies). Particular care was taken on the selection of the primers based on low background signal that is crucial for the unambiguous assessment of length and point heteroplasmy. Moreover, we kept the complexity of the laboratory process as low as possible. This was achieved by a 96- well based pipetting format for cycle sequencing set-up using only two PCR amplicons per sample which reduces the chances of contamination, handling error and sample mix-up.

## Methods

### Samples

A total of 10 biopsy samples were collected from patients with diagnosed invasive mamma carcinoma at the Clinical Department of Radiology, Innsbruck Medical University. They were immediately transferred into 1.5 mL vials and extracted or frozen at -20°C and extracted within the next 7 days. Corresponding blood samples were collected independently. Peripheral blood samples were either extracted upon receipt or frozen at -20°C and then processed within 7 days. There was full consent of the patients to participate in this study. For the sensitivity study DNA was extracted from peripheral blood of a healthy volunteer of European ancestry with informed consent.

### DNA Extraction

The complete workflow is sketched in Figure 1. DNA was extracted from biopsies and blood via the EZ1 Biorobot workstation (Qiagen, Hilden, Germany). Extraction was based on the protocol of the investigator kit (Qiagen) for extraction of blood and tissue samples, with modifications as follows: tissue samples (about 0.5 cm × 1 mm) were digested with 15 μL proteinase K in 290 μL lysis buffer (included in the kit) up to 4 hours and treated with the "trace" protocol.

### Primer Design

A total of 96 primers were used for singleplex reactions. Primer sequences were taken from [29,33] and new primers were designed in this study with PRIMER 3 software [34]. Possible heteroduplex formations, primer dimers and hairpin structures were analyzed with OligoAnalyzer free software:  (Integrated DNA Technologies, Coralville, IA). Melting temperature of primers was approximately 50°C and the GC-content was between 40 – 60%. The settings for the calculations were: Oligo conc. n: 0.25 μM; Na^+ ^conc.: 50 mM.

### Amplification and Sequencing

For amplification we used the Advantage GC Genomic LA Polymerase(Clontech, Bella Avenue Mountain View, California) including a small amount of proofreading enzyme, a hot start antibody and a 3' to 5' proofreading exonuclease activity. It enables synthesis of PCR products of 8.5 kb using human genomic DNA templates.

Amplification reaction was carried out on a thermal cycler (Multicycler PTC240 Tetrad2, Hercules, CA) in a total volume of 50 μL each fragment (A and B) comprising 2.5 mg/mL BSA (St. Louis, Missouri), 2.5 mM each dNTP (Applied Biosystems), 10 mM each primer, 5U LA Genomic DNA polymerase (Clonetech) 1000 – 10000 mtDNA GEs. Thermal cycling conditions comprised an initial 3 min denaturation step at 93°C, followed by 93°C for 15 s, 60°C for 30 s and 68°C for 5 min 14 times proceeding 27 times with 93°C for 15 s, 55°C for 30 s and 68°C for 9 min increasing for 10 s each cycle. Amplicons were purified from residual primers and dNTPs enzymatically with ExoSAP-IT (ExoSAP-IT, USB, Cleveland, Ohio) and comparatively with a filtration method (Microcon YM-30 Centrifugal Filter Units: Millipore, Billerica, Massachusetts).

From a 1.5 μM stock plate containing all 96 sequencing primers (Figure 1, step 4), 2 μL were decanted into a new plate right before each sequencing reaction. Big Dye Terminator mastermix (containing 1 μL BigDye Terminator v1.1 Cycle Sequencing mix (Applied Biosystems) and 3 μL BigDye Terminator v1.1 Sequencing Buffer (Applied Biosystems)), as well as 2 μL mtDNA Fragments A or B were aliquoted into the appropriate wells as shown in Figure 1 (step 4). 2 μL distilled water was added to reach a final volume of 10 μL. Pipetting those small volumes was performed using an 8-channel epMotion workstation (Eppendorf AG, Hamburg, Germany). Cycle sequencing was performed (after a first denaturation step of 95°C for 1 min) for 25 cycles of 10 s at 95°C, 5 s at 50°C, and 4 min at 60°C.

### Post sequencing cleanup

Sequencing reaction products were purified from residual dye terminators using Sephadex G-50 Fine (Amersham, Buckinghamshire, United Kingdom) and Multiscreen filter plates (Millipore) according to the manufacturer's protocol. The cycle sequencing products were diluted by adding 10 μL of distilled water and the dilutions were centrifuged through the filter plate into an optical 96-well plate for electrophoretic separation. The entire procedure of diluting cycle sequencing products and transferring the dilutions onto the Sephadex columns in the filter plate was again performed by the epMotion workstation. When spinning cycle sequencing products through the filter plate, unequal amounts of product may be recovered throughout the plate. In order to avoid this, the blocks' orientations in the centrifuge carriage were reversed after 2.5 min and the blocks were spun a second time for 2.5 min to obtain consistent amounts of purified products. The purified products were finally diluted by adding each 20 μL of distilled water to achieve volumes of 45 μL.

### Capillary Electrophoresis

Electrophoretic separation was carried out on an ABI 3100 capillary sequencer (Applied Biosystems) using POP6 and a 36 cm capillary array. The run module conditions were as follows: injection time: 22 s, injection voltage: 1 kV, run voltage: 15 kV, run current: 10 μAmps, run temperature: 55°C.

## Authors' contributions

LF developed the whole mt genome sequencing strategy, coordinated the experiments and drafted the manuscript. BZ and MD performed experiments. WP participated in study design, supervision, and revision of the manuscript. All authors read and approved the final manuscript.
